# The Corn-Stalk Disease in Cattle

**Published:** 1889-09

**Authors:** Frank S. Billings

**Affiliations:** Director of the Patho-Biological Laboratory of the University of Nebraska; Lincoln, Neb.


Buffalo Medical and Surgical Journal

VOL. XXIX. SEPTEMBER, 1889. NO. 2.

### Original Communications.

### THE CORN-STALK DISEASE IN CATTLE.

BY FRANK S. BILLINGS,

Director of the Patho-Biological Laboratory of the University of Nebraska.

(*Concluded.*)

### MORPHO-BIOLOGICAL CORRESPONDENCE BETWEEN TWO OR MORE MICRO-ETIOLOGICAL ORGANISMS NOT SUFFICIENT GROUNDS FOR PRONOUNCING THE DISEASES WITH WHICH THEY ARE CONNECTED IDENTICAL.

THE details of this discussion will be found in my report on the swine-plague. It is necessary, however, to touch upon the essential points here also. As was there shown, Hueppe asserts that the European diseases previously mentioned as being caused by a member of this group of belted, ovoid germs, viz., the "Hühne Cholera, Kaninchen Septikæmie, und Wild Seuche" are all one and the same disease, because their micro-etiological organisms have the same form, the same size, the same belted appearance, and because they all grow alike in bouillon, upon agar-agar, and in beef-infusion gelatine.

No greater or more misleading and illogical pathological misconception could possibly be made, than this statement of Hueppe with regard to the diseases named.

It may be axiomatically asserted that the most complete morphological resemblance, and exact morpho-biological resemblances, in or upon any artificial media, are not sufficient grounds for any such generalization in classification of diseases as that attempted by Hueppe.

To all beginners in the work of patho-bacteriology, and to all older hands as well, I most dogmatically assert that there is but one factor in the biology of micro-etiological organisms which can decide whether two apparently alike germs are one and the same object when derived from two distinct diseases of animal life. That factor is a physio-chemico-biological one! The character of the ptomaine produced, or the lesions resulting in inoculated animals, decides it. Both germs must produce the same disease in both species of animals, the same chemical and pathological phenomena which occur in the same diseases and in the same species of animals under natural conditions, when healthy animals of the given species are inoculated with artificial cultivations of the germs in question.

Our experiences here completely upset Hueppe's hypothesis!

The American swine, the southern cattle-plague, the yellow-fever, and the corn-stalk disease should, according to Hueppe, be identical diseases with those mentioned as such by him in Germany; because, according to his conditions, the germs are identical in appearance. Hueppe's entire argument is completely nullified by the following facts:

First—There is no southern cattle-plague known in Europe.

Second—Cattle and swine run together in this country, and one or the other may have respectively swine or cattle-plague, and yet the other species will never become ill, even from the closest contact with members of the other species ill with its peculiar plague; also in the corn-stalk disease.

Third—The same is true of hen-cholera and swine-plague in this country. Hens can feed on hogs dead from swine-plague, from the ground polluted with their discharges, even picking out grain from the same, and still remain well, and the same is true of the hogs with regard to hen-cholera and the southern cattle-plague.

Hence, no matter how germs may resemble each other when artificially examined, they fail in the one great factor necessary to make the diseases produced by them identical.

Fourth—Human beings do not have the yellow-fever at the same time cattle have the southern cattle-plague in the same locality. They do not have the same physiological chemical attribute with regard to a given something produced, which invariably decides the pathogenic results produced by a given germ. Notwithstanding these facts, these diseases have a very close relation to one another.

They are all extra-organismal, local, land septicemiæ. Each one, however, has something peculiar about it that prevents it from being identical with the others, aside from any action of the germ itself. It is this: each species of animals in which they produce a specific disease has some unknown constitutional idiosyncrasy which renders its members susceptible to the action of a given germ, and each of these germs has some peculiar unknown biological idiosyncrasy by which alone it infects naturally but a given species of animal life. These two factors together can alone decide the identical question. What we can do artificially, by the inoculation of other animals than the disease occurs in naturally, has no necessary relation to the question whatever.

To return to our subject.

There are, however, other phases in the development of these germs of a bio-morphological character. For instance, as already said, we may see two or three individuals, of the mature type, united together (Fig. 2), or we may find two apparently mature organisms enclosed in a common capsule, the two medial dark points or poles being in such close apposition that no line of demarkation or indentation of the capsule can be seen at this point, the whole outer surface being smooth (Fig. 7); on the other hand, the two lateral ends or free poles are separated by the normal quantity of white non-colorable substance. Again, these diplo-bacteria may assume a curved or sausage shape, which we may sometimes see intimated in the single organisms (mature—Fig. 8). At other times, though not very frequently, the germ may appear in a nearly normal form, but one pole- (coccoid) end will be semi-segmented from its appositional end of the white substance by a constriction of the same at its line of attachment with the pole-end (Fig. 9). This end will then be smaller than the opposite pole, thus giving a sort of pear-shape to the entire organism; the small pole-end is soon dropped, however, and becomes momentarily a free coccoid, and goes through the cycle of morpho-development already described; the same occurs with the other pole-end. This concludes my observations of the micro-morphological phases presented by these two etiological organisms in the course of their development. There may be some minor phenomena that have escaped my attention, but I am very sure that I have described all the essential points.

### ESSENTIAL CHARACTERISTICS OF THE DEVELOPMENT OF THE CORN-STALK DISEASE ORGANISM AND THE POINTS OF DIFFERENTIATION BETWEEN IT AND THOSE OF SWINE-PLAGUE AND SOUTHERN CATTLE-PLAGUE IN AND UPON DIFFERENT CULTIVATING MEDIA.

The germ grows well and most characteristically at ordinary room temperature.

*Potatoes.*—Upon the cut surface of sterilized and steamed potatoes the germ of the corn-stalk disease grows as greyish-white somewhat elevated colonies, while that of swine-plague develops in a sort of yellowish, dirty, olive color, somewhat resembling muddy coffee, and that of the southern cattle-plague in primarily straw-yellow color, which eventually assumes a slightly reddish shade. Very soft, moist potatoes will affect the growth and its color.

*White of Eggs.*—The most practical way of sterilizing this material and getting it in a convenient form, is to steam the eggs until cooked hard, and then remove the requisite portion of the shell with sterilized forceps, after which they are placed in a moist chamber, prepared in the same manner as for potatoes or plate cultures.

Upon this medium the corn-stalk germ grows as a clear yellow colony, with slightly raised edges, and is to be easily distinguished from that of swine-plague, which develops as a somewhat opal, projecting, white, semi-fluid mass, the center being the much more prominent, and that of the southern cattle-plague, which presents a buff-colored colony.

*Agar-agar* (plain, not glycerine).—Upon this medium, when the surface is dry and not accidentally moistened by the fluid in the bottom of the tube, the corn-stalk germ develops in a really characteristic manner, in contradistinction to the other two. In the color of the development there is nothing essentially different to be seen, but, in comparison with the swine-plague organism, the edges of the cultures are more distinctly scalloped, the separating lines extending deeper into the body of the culture; but a marked point of difference is, that, commencing from the line of inoculation, the corn-stalk germs form lines or rays which are finally limited by a diffuse semi-transparent border. Each of these lines is made up of individual colonies; again, the culture is much more dry and less viscid than that of swine-plague (which is of a diffuse greyish color), and adheres much more closely to the underlying agar; in fact, when old, it will break up in fragments on attempts at removal with the wire. As said, in order to get these effects, the agar must be just right, and have a dry surface. On agar, there are no essential points of difference between the swine-plague and southern cattle-plague organisms, except rapidity of development and a slight variance in the irregularity of the edges of the cultures.

*Beef Infusion Gelatine Punctures.*—The growth in gelatine itself, of these three organisms, offers nothing of a differential, characteristic nature, but their development upon the surface varies considerably, the germ of the corn-stalk disease developing and spreading of the surface the most rapidly, and with more leaf-like extensions. It is also more dry and lusterless than either of the others. That of the southern cattle-plague is next in rapidity of development, while that of the swine-plague develops the more slowly, and has the more prominent edges. Both this and that of the southern cattle-plague form a more or less viscid colony, while the corn-germ adheres to the gelatine, and is inclined to break up on removal with the wire, especially if the cultures are a little old.

*Oblique Surface of Beef Infusion, Gelatine.*—As neither of these organisms cause this medium to become fluid, it was quite interesting to see how they would grow on gelatine prepared in this way. The germ of swine-plague here develops, or shows, more of a yellowish-white shade than the others, and has a greater degree of opacity, the edges of the growth being very delicately irregular. The corn-germ extends the most rapidly and the growth is more pellucid, pearly-white, than the others, the edges being much more deeply scolloped, and towards the bottom of the growth a delicate, smooth, pearly-white edge embraces the scollops; the southern cattle-plague germ, the most slowly of all, being of a milky-white color, but less intense than that of swine plague. The edges of the growth are more delicately irregular than in either of the others.

INOCULATION EXPERIMENTS WITH PURE CULTURES OF THE CORN-STALK ORGANISM.

The next step was to prove the malignity of the pure cultures thus obtained. To this end there were inoculated, February 11, 1889, one full-grown buck rabbit, with a pure bouillon culture; in sub-cutis of right ear, two drops; in that of the inside of right flank, three divisions of a 1 c. cm. syringe. The ear was inoculated, especially to see if there would any extreme degree of tumefaction follow.

2. One male, full-grown Guinea-pig; three divisions of a syri ge inside of right flank.

3. Mouse; one drop in same locality.

Morning of 12th, rabbit ill; will not move unless made to; not eating; no local tumefaction. Mouse very ill, coiled up in corner of cage, and only moved on disturbance. guinea-pig same, but not so much depressed; nibbling a little food.

12th, 3 P. M.—Mouse just died. No edema at point of inoculation, but local vessels engorged; mesenteric vessels engorged, and serosa of abdominal cavity swollen and glistening; muscles looked as if cooked; spleen, liver, and kidneys swollen and very full of blood; lungs congested.

12th, 3.25 P. M.—Rabbit dead. Skin and subcutaneous tissue at locus inoculationis of flank much engorged, but no edema present; of a diffuse red color, with marked injection of larger vessels; no effect in ear other than mentioned; no effusion in cavities of the body; blood extremely thin and of a purple-red color; lymph-glands much swollen and very juicy. *Liver* swollen; serosa normal, but the parenchyma reflecting through it was of a yellowish grey-red color; peripheries of acini yellowish grey; center red; cut surface very friable, opaque, and anemic, yellowish grey in color. *Kidneys* swollen; capsule non-adherent, cortex anemic, opaque grey-red in color; medullary substance of a diffuse dark-red color. *Spleen* swollen; pulp very juicy and soft; vessels of mesenterium engorged, those on curvatures of stomach the same; mucosa of stomach swollen, covered with thick viscid coating, diffuse bright-red in color; contents of small intestine fluid, mucosa much swollen, yellowish red in color, and covered with a viscid coating. Contents of large intestine pultaceous; mucosa not swollen. Lungs engorged. Pure culture of same germ as found in material from the Fremont animal derived from each organ, and accurately tested by comparison with original culture as well as microscopically. Same organism in small intestines and isolated from the others there by plate cultures.

13th, 2 P. M.—Guinea-pig dead. Lesions same as in rabbit. Cultures conformable.

These experiments demonstrated three facts of great differential diagnostic value. Aside from those demonstrated by the cultivation experiments, these inoculations showed that the organism in question could not be that of swine-plague, on account of its acute fatality. It could not be that of the southern cattle-plague, because of the immunity of rabbits to that germ in such small doses. It could not be the germ of the German "Wild-seuche," because of the total absence of the enormous edema which invariably follows such inoculations in that disease.

It must, then, be a new disease!

EFFECTS OF INOCULATIONS WITH THE SAME MATERIAL IN A STEER AND HOG.

These inoculations were made on the 9th, two days previous to those in the small animals mentioned, six c. cm's of a pure bouillon culture being injected under the cutis in each case. The hog was not affected at all, and with that we will leave it.

Steer-calf, five months old.

10th—Temperature, 10 A. M., 39.50° C.

11th—Temperature, 10 A. M., 39.50° C.

12th—Temperature, 10 A. M., 39.70° C.

Locus inoculationis slighly swollen and hot; animal lying down, loth to move; respiration 60 per minute, pulse much accelerated; right lung solidified for the lower half; augmented vesicular respiration, with some thickening of the sound in superior half of this and whole of left lung; drinking much, but eating little.

13th, 9 A. M.—Temperature 41° C, respiration 60, pulse as before; made to rise with difficulty; right lung still more solidified, some pleuritis in lower portion; eyes wild, conjunctiva injected, yellowish material escaping from nostrils; severely constipated, feces dark and hard, urine albuminous.

13th, 5 P. M.—Temperature 40.50° C.; manure becoming pultaceous; otherwise same.

14th, 9 A. M.—Temperature 40.70° C.; condition about same; animal emaciating rapidly; discharge quite fluid and of a yellowish color.

15th, 9 A. M.—Temperature 40.70° C.; manure a little more solid, respiration somewhat easier; animal eating a little; lung clearing up somewhat.

16th, morning—Temperature 39.90° C. Evening, 39° C.; animal improving.

17th, morning—Temperature 36.2° C. Evening, 36.2° C.

This looked like death. The animal was shivering, and terribly emaciated, and I felt sure of an autopsy in the morning, but on the

18th, morning—Temperature 37° C. Evening. 37° C.

19th, morning—Temperature 37° C. Evening, 37.1° C.

20th, morning—Temperature 37.1° C.

The animal eventually recovered, though it became excessively emaciated. On the 15th, I washed the inferior surface of its tail with cor. sub. 1.1000, and with a sterilized knife opened the local vein, allowing the blood to flow directly into a sterilized homeopathic vial. From the same I again obtained pure cultures of the germ, which were proven by cultivation comparisons with the various others, all of which were kept up on the different media mentioned until the investigations were terminated to my satisfaction.

### A FEEDING EXPERIMENT WITH PURE CULTURES.

Fed a full-grown female rabbit with pure bouillon cultures from the Fremont cow, by pouring 25 c. cm's. between the leaves of a quarter of the head of a cabbage two days in succession, beginning with February 14th. 15th and 16th, no change; eats with avidity. 17th, somewhat quiet, but still eats its rations pretty well. 17th, back arched, hair bristling, sitting quiet in corner of cage; respiration somewhat increased, eating but little. 18th, not eating at all; remains in one place, back arched, respiration very rapid. 19th, 9.30 A. M., dead in cage on arrival at laboratory. Somewhat emaciated, musculature very pale, peritoneum swollen and diffuse pink-red with occasional punctiform red spots scattered through it; serosa of the stomach and small intestine swollen and clouded; vessels much engorged. Liver swollen, of a mottled greyish yellow and red color. Gall-bladder distended; duct open into intestine; outside surface anemic, opaque, and yellowish grey-red in color. Acini swollen; periphery yellowish grey in color and glistening in character, center reddish. Spleen excessively swollen, pulp soft and juicy; kidney swollen; cortex opaque and anemic, yellowish grey in color, diffuse red. Thoracic cavity contained about a tablespoon of yellowish red fluid; right lung attached to ribs in all directions, and solid throughout. Pericardial sac totally obliterated; portions of left lung solid, and red centers throughout solidified parts. Urine albuminous. Cultures from heart's blood, spleen, liver, lungs, and kidneys contained the same germ as in previous cases, and developed in the same manner.

### A FEEDING EXPERIMENT WITH CORN-FODDER FROM FREMONT, NEB.

Immediately upon finding the same microörganisms in the blood and organs of the cow killed at Fremont, that I had found in the same kind of material from Ames and Cortland the previous year, and feeling there might be a hidden truth behind the "dry corn-stalks" theory, I wrote to Mr. Delaney, of Fremont, to send me some of his corn-stalks, and to strip off the leaves, so as to save bulk. He very fortunately misunderstood me, and sent a small package of the stripped leaves, totally insufficient for any feeding experiment in cattle. On account of extreme demands on my time, I let the material remain in the package, and gave no thought to it until February 26, 1889, when Dr. E. O. Shakespeare, of Philadelphia, being here, and wanting a piece of paper to wrap up something in, I undid the corn-leaves and gave him the paper. As a mere matter of curiosity, I gave the leaves to a rabbit to eat, cutting off all other food, but giving it an abundance of fresh water twice daily. So little did I think that the leaves would lead to any positive effects in the rabbit, that I gave no attention to the animal until my man told me it was dead, on the morning of March 4th. In fact, it had just died as we got to the laboratory. It had been eating green food for the past two days, but had not touched that given it on the third. My interest in the matter was very great, and no time was lost in making examinations and cultures. In the blood, liver, spleen, kidneys, and mesenteric lymph-glands, the same germ as that found in the cattle was present, so far as one could judge from a microscopical examination. Cultivation experiments gave all the points of differentiation which experience has shown can be relied upon. The cultures were all strictly pure, as shown by cultivations on potatoes, eggs, and in gelatine.

The lesions in the rabbit were briefly these: Swelling of the peritoneum, with straw-colored effusion in cavity. Liver excessively swollen, very friable, and almost a phosphorous liver in portions, in its degree of fatty degeneration. Spleen same as to swelling, pulp almost semi-fluid. Kidneys, cortex almost pure yellowish grey, opaque, and anemic; friable also. Stomach two-thirds full, mucosa intensely swollen, covered with a thick, viscid coating, beneath which the tissues were a diffuse purple-red. Contents of small intestine fluid, mucosa very thick and swollen, covered with a glary viscid coating, and anemic, as in the feeding experiments with the swine-plague germ. Large intestine, contents pultaceous, mucosa but little swollen though much injected, mesenterial lymph-gland resembled the strawberry-like glands seen in hog-cholera; excessively swollen; vessels of membrane engorged. Thoracic cavity, heart-muscles opaque, very anemic, and friable; bronchial lymph-glands diffusely red and juicy; left lung hyperemic; right, normal.

Thus, it will be seen, I have followed this germ, first finding it in the cattle, and then tracing it from them to the corn, demonstrated its malignancy in that material; and beyond that, the accidental sending me of the leaves, instead of the stalks, by Mr. Delaney, and the positive result in the rabbit fed with those leaves, closely conforms to the practical fact that cattle, when turned into such stalk-fields, naturally take the leaves and tender top-shoots, leaving the hard dry stalks more or less untouched, and hence it is from them that they become infected. I believe this to be the first actual demonstration of a germ of a malignant nature infecting our grasses or grains during their development, and having disease-producing properties for certain forms of animal life when fed upon them. The question still remains open, so far as this germ is concerned: Does it also penetrate the ear? That it is in the stalks, I have myself demonstrated by microscopical examinations and cultures, but with this singular result: *It is not disease-producing when inoculated in either rabbits or mice from this source, while from the leaves it is, as we have seen from the feeding experiments in the black rabbit.*

This fact again opens up a still more interesting question, viz.: *Does the nature of the soil (nutrition), or the chemical properties in which these extra-organismal infectious elements primarily live, or into which they get, cause their specific infectiousness in relation to animal life?*

We do know that the chlorophyl, and perhaps some other elements in this case, of the leaves of the corn has a different chemical composition from the pith of the stalk or even its woody covering, unless it be its extremely superficial layer; hence, we must face the question:

As this organism is not disease-producing when derived from the stalks, does the germ cause a chemical decomposition in the elements, juices, present in the leaves, or does chlorophyl supply a material which causes such changes in the physiological attributes of the germs as make them disease-producing for a certain length of time? It is well known that in artificial media they either lose their infectious property in time, or can be made to do it at our pleasure. In fact, we are able to graduate that property at will. As to the swine-plague germ, I have experimentally demonstrated that a mitigated culture of these germs, which will not seriously affect a bunch of pigs when inoculated with 1 c. cm. under the cutis, though sufficient to protect them from the most severe and repeated exposures, still gets into the contents of the intestines of the inoculated animals, and when passed off in their manure, and remains there for a time under conformable conditions of temperature, becomes excessively virulent towards healthy hogs if put where such manure is, their food being scattered among it, so that they are obliged to consume more or less of such infected manure as they seek their food.

Again, we have to discover whether this corn-germ also infects grasses and other fodders, such as millet, as is rather indefinitely indicated in the letter from Dr. Brayton regarding the outbreak at Cortland, Neb., to which reference has been made.

These are all questions of immense hygienic importance to our stock-raisers, and perhaps to man as well. For, should it be demonstrated that the ear of corn is dangerous also, we have to face the question: Is it in the grain also, and is a meal from such grain dangerous to humanity as food? For, in these things, we cannot always depend on cooking, though probably we can in this case, as this corn-organism is not spore-bearing.

The pathological investigator cannot settle this corn-disease question altogether. Having traced it directly to the corn, and demonstrated the fatality of "dry murrain," impacted third stomach, corn-smut, insufficient water-and-salt, and what-not absurd theories as to the origin of this disease in cattle, it now remains for the botanist, or perhaps more truly, botanical mycologist—the simple pure bacteriologist—to trace the organism in corn, and tell us or, better, the farmers, how it affects corn, and how they can distinguish such corn—for the disease has its language in corn—so that the feeder will be enabled, in the near future, to avoid using such fodder in feeding his cattle and horses. One thing is very certain; this corn-disease is not only exceedingly local in its extension, being not only limited to certain fields, but even in certain portions of such fields, and, most probably, to certain stalks in a hill, or, at least, to individual groups of stalks.

Some valuable and interesting testimony upon this subject is given in the following letters from my esteemed friend, Prof. Burrill. From them it will be seen that Prof. Burrill should be accredited with the first real discovery of this interesting and, unfortunately, fatal organism to our live-stock. But, as Priscilla said to Alden, "Prithee speak for thyself, John":

UNITED STATES BOARD OF INQUIRY CONCERNING EPIZOOTIC DISEASES OF SWINE.

CHAMPAIGN, Ill., February 14, 1889.

*My Dear Doctor*—The disease you describe in cattle is a common one in this vicinity in the Fall and early Winter, when the animals are turned into the corn-stalk fields, and has occurred, I think, under other conditions. The veterinarians have pronounced it due to an impaction of the third stomach, just as your men have. I have not myself seen a *post mortem*. The thing is of much interest, practically and scientifically, and I will look out for it.

My best regards to you, and believe me, very truly yours,

T. J. BURRILL.

CHAMPAIGN, Ill., March 11, 1889.

*My Dear Doctor*—I find, on comparison with my old slides, that the microbe from your cultures differs mainly in its smaller size. I send you a slide taken from the diseased corn-stalk (maize). This was two years ago. I did not deem the matter sufficiently worked up to publish, but did publish the account of the organism on broom-corn and sorghum, a different thing from this on maize. This corn-stalk trouble is, as I found it, very local, occurring in a given area of a field, and not elsewhere. In several instances I found it only upon low spots, and in two instances on ground that had been pond-holes until the year before, when they were tile-drained. In another case, a whole field of ten acres, on rather sandy soil, and in clover the year before, was affected. Last year, a man sent me some green stalks which he said came from a forty-acre lot, nearly all of which failed, though soil and season seemed favorable. The stalks showed plainly enough the special trouble of which I now write. The most marked effect is to be noted in the leaf-sheaths, and if you can get a culture at all from the corn-stalks, I think you can best do it by stripping off some of these sheaths and taking material from the inner surface of the tissue in corroded spots. Your cultures all seem pure and, as I have now some young corn growing, I will try the effects of the same upon it. Your germ is motile in liquids, and in this agrees with my corn microbe. Neither liquifies gelatine. But I did not sufficiently study the thing at the time to be able to compare much further.

Yours, BURRILL.

Upon examining the slide sent to me by Prof. Burrill, I find the difference in size mainly due to the fact that he used a blue tincture to color his germs, which, with this organism, gives a diffuse washy outline, thus making it look larger than when colored with fuchsin, when its outlines are very sharply marked. However, I have no doubts but that my testimony and that of Prof. Burrill will eventually completely coalesce; though being an infinitely more skilful bacteriologist than I pretend to be, being but an embryo pathologist, I do not doubt that he may not only find something to correct, or differ from, in my bacteriological work, but add much interest and value to it, though not militating at all against its general correctness. My interest in bacteriology does not extend an iota beyond the relation of specific germs to the diseases caused by them, and such points as are directly essential to the differential diagnosis of one germ from another, but only as they bear upon disease. A bacteriologist I am not, and have no interest whatever in adventitious germs, or bacteriology *per se*.

The following letter from Prof. Burrill is still more interesting and valuable:

CHAMPAIGN, Ill., May 1, 1889.

DR. F. S. BILLINGS, Lincoln, Neb.:

*Dear Sir*—According to your request, I herewith send you some account of a disease affecting growing corn (maize) in the field. It must be premised, however, that the malady, as affecting this staple crop, has not been fully worked out, because, for some unknown reason, the attempts to communicate the disease to healthy plants by the application of culture materials containing the living organism derived from diseased plants have not been successful. I am thoroughly assured, however, that these failures are from some fault in the methods tried, rather than in the want of having the true disease "germ," for a specific organism is too uniformly present in the affected tissue to permit us to consider it accidentally connected therewith. Moreover, the progress of the disease from cell to cell corresponds exactly with the spread of the minute organism in these tissues. It is never found far from cells which plainly show the disease characteristics, though I have succeeded in making cultures from parts appearing healthy upon the very border of the affected portions. But this only goes to show that the healthy cells are invaded before they present the characteristics of the diseased cells; that is, the organism causes the disease, and not the disease the organism. There was a time, perhaps, when this latter alternative might have been seriously considered, but if so, surely the time has gone by. While no one will rest content with the simple presence of a microbe even constantly in affected tissue, as full and conclusive proof that such microbe is the actual active agent in the disease, still it must be admitted that this is exceedingly strong evidence of such active agency. In the present instance, I have little hesitation in saying, without successful inoculations, that the organism which has been so often found in the diseased parts of the corn-plant, and which has repeatedly been obtained in a state of purity by cultivation methods, is the direct cause of the mischief observed. I am the more confident of this from the fact that but few inoculation experiments under natural conditions have been tried. Of course, continued failure of these last would shake the confidence now felt, but up to the time of this writing there is no cause for such skepticism. It should be said that, although some work was done upon the disease during the two seasons of 1887 and 1888, other duties were permitted to crowd out this special investigation, except as stated below. Further, it must be said that, so far as I now know, the evidence that the organism found by me in diseased corn is identical with that sent me by yourself in cultures from rabbits dead from eating suspected corn-stalks, rests entirely upon their microscopical appearances. I had only mounted slides of the former for comparison, the cultures having been lost. Under the microscope they do seem to be identical. Both are actively motile, as my notes of the former indicate for that direct from the corn, and as is readily seen in the preparations of the living "germs" from the rabbits. Neither, it appears, produces spores—at least, endspores. They behave the same in staining characteristics, so far as tried by me. Now for the notes you request.

The disease in the growing corn commences at any time during the warm season. According to my observations, it is most likely to become noticeably apparent after mid-summer, or after the corn "shoots," though this is by no means always the case. Very often it occurs only upon certain pretty clearly marked areas, or patches, in the field. I think this must be very generally the case, for only two exceptions have fallen under my observation. One of these exceptions was a field of about forty acres, the other of less size; both had fertile soil, and were well cared for. Neither paid the expenses of cultivation. As generally found, the affected patches are easily recognized. The corn fails to grow as in the healthy areas. Its stunted size at once arrests the attention. Diseased stalks may, indeed, be found as large as the largest, but it is probable that these became later affected. In one instance, an area of about an acre, planted with the rest, did finely until the young corn was about six to eight inches high, after which it died so completely that the farmer replanted the patch. This latter planting did well, so far as is known.

Along with dwarfish appearance, designated as common, the lower leaves prematurely die, passing through the stages of becoming yellowish green, then yellow, then withering away. Upon close examination, it will usually be seen that there are certain spots, more especially upon the basal part of the leaf, which is wrapped closely around the stalk, having a different discoloration. These are brown, watery-looking at first, then dark, and finally dead. Occasionally there are livid red spots or patches in the same situation, but I am not sure that these mean the same thing. These specially affected spots vary in size from mere points to those of several inches across, often larger in the direction of the veins of the leaf or leaf-sheath. It is in such diseased parts that the microscopic organism believed to cause the trouble can always be easily found, and from which cultures are readily made in beef-broth, the juice of the corn-stalks, etc.

The remaining characteristic of the disease is really the one most conspicuous to those who search carefully. When the corn suffers worst from the malady in question, the roots are badly affected. Beginning with the oldest and lowest, they die and decay in the ground. At length the stalk is held upright only by the later-developed "brace" roots, and even these may slowly corrode away. Under such circumstances, the affected stalks are very easily pushed over or pulled up from the little hold they have in the ground. If the plant is carefully dug, and the affected roots examined, even with a magnifier, no evidence can be found of the work of worms, or of insects of any kind. The roots simply die, though no wounds are to be found. The external layer rots most easily and quickly, and the woody inner part may then be pulled out like a string.

Of course, when the roots are thus affected, the whole plant suffers, ceases to grow, fails to mature its ear. Anyone and everyone, who observes it at all, knows that something is the matter. If one now looks closely at the brown spots on the leaf-sheaths or roots during the first stage of the disease, he will often find little collections of gelatinous-like exudation. Crush a minute bit of this under a microscopical cover-glass, and examine with a high power of the compound microscope, and the living organism to which we ascribe the disease can be seen in innumerable numbers.

So far as observed, corn on rich land is more likely to suffer, not unfrequently that in low places recently broken up from the sod of wild grasses seems to be the worst affected. Sometimes, instead of distinct areas in a field being alone injured, scattered stalks throughout the plantation are diseased, but this seems much less common.

The corn disease, as now described, is very similar to that affecting broom-corn and sorghum, but is, nevertheless, due to a distinct organism, having at least some differences in mode of development and action under different circumstances. The broom-corn disease was deemed sufficiently understood for publication in 1887. The topic was presented by me to the meeting at Cleveland, Ohio, of the Society for the Promotion of Agricultural Science, and the paper was published in the proceedings for that year. It was also published in the Transactions of the American Society of Microscopists for the same year.

I am, very truly yours, T. J. BURRILL.

### NATURE OF THE CORN-FODDER DISEASE.

It is not necessary to go into a very detailed discussion of this point, as it has been sufficiently treated in the preceding article upon the southern cattle-plague, Part III., of my report, and even more fully in my report upon the swine-plague of the United States.[1]

Like both of those pests which so seriously interfere with the prosperity of the live-stock interests of the United States, this "corn-fodder" disease has nothing of a contagious character about it. It does not owe its primary origin to the presence of a diseased animal in the first place, or of any material from such a diseased animal among healthy stock. It is not an endogenous, generating from within, disease; on the contrary, a better example of an exogenous, owing its genesis or origin to external circumstances, disease, could not possibly be found. It most aptly illustrates my endeavors to impress upon the medical world the absurd folly of the continued use of the words contagious and infectious in any endeavor to express class in disease. The word "contagious" simply expresses to us the fact that the infection of a healthy individual, in a given disease, either took or can take place by means of contact either with an individual diseased with such a specific disease, or with some material directly from such an individual. Correctly speaking, then, the word contagious only means that the primary origin of the inficiens—infecting material—was within the body of an already diseased animal. Any other definition of this word is absurd, but the term which I have selected to express this class of diseases, while equally as technical as that of Pettenkofer—"Endogenous"—is more self-evident to the ordinary class of readers, viz., "*intra-organismal*," that is, originating within or from a diseased animal organism. On the contrary, I have called the opposite class "*extra-organismal*" diseases, simply because their

1. See review, p. 224, Vol. XXVIII., this JOURNAL.—ED.

primary origin must invariably be sought in external surroundings or conditions.

In the disease in question, we have already demonstrated that its primary cause is to be sought in the corn-fodder, and perhaps the grass which certain susceptible animals eat.

Again, this disease bears a very close resemblance to both the southern cattle-plague and the swine-plague, in that it is an absolutely local disease. Mr. McKelvie's letter demonstrates that fact, in optima forma, for he tells us that it was the corn-fodder in a certain field which was dangerous to his cattle, and all practical experience in every part of this country serves only to confirm that statement without even a single contradiction. Professor Burrill tells us the same thing with regard to the disease in the corn itself, and I feel very sure that more exact study will eventually demonstrate that, in many cases, this localization, with reference to the disease in corn, will be found so centralized that even a single stalk in a single hill or a group of stalks, will alone be found diseased, while in another there will be several, and in still other places whole groups of stalks, or even portions of a field in the major part of it, will be complicated, as witnessed by the "forty-acre" field quoted by Prof. Burrill.

### THE CORN-FODDER DISEASE A SEPTICEMIA.

Were I to be asked what disease of animal life this corn-fodder disease most closely resembles, I should say it is the exact counterpart of the genuine swine-plague, and, in fact, most respectfully refer the reader to page 320 of that report, upon "the intra-vital phenomena" in that disease. In the corn-fodder disease in cattle, every organic lesion and every variety of lesion seen in the swine-plague will be seen with the exception of the ulcerative or neoplastic lesions and perhaps diptheritic, seen in the intestines, which are primarily due to idiosyncrasies of structure in the hog and only secondarily to the action of the bacteria causing that disease. Here, too, we have the same excessive parenchymatous changes in the great glandular organs of the body common to all acute diseases of this character. Here, too, we have pneumonia in all the various types as in swine-plague, and again one form bearing the very closest resemblance to the same in swine-plague worked by the engorgement of the inter-lobular vessels and coagulation of the blood within them, and which is equally well applicable to this corn-fodder disease. In both species of animals, the pregnancy of this lesion is due to the peculiar loose and open character of the interlobular tissue and the large vessels circulating in this tissue, which is common to both species of animals. Again, in both diseases we find an acute broncho-pneumonia, not due to the entrance of the specific germs via the respiratory tract, but to the extreme degree of interference which the circulation suffers and the consequent effusion of serum into the air-tubes, especially the smaller, obstructing them and leading to atelectasis and pneumonia. I neglected to call attention to this variety of consolidation in my report on swine-plague, but it is really a very common form and to be easily distinguished from that variety caused by the entrance of the germs via the respiratory tract, *which is more acute, more multiple, more rapidly caseous and especially not hemorrhagic in any of its parts or in the surrounding tissue.* This is a point which I would especially call to the attention of some investigators in this country who fondly imagine themselves authorities upon questions of pathology, especially that of swine-plague.

In the corn-stalk disease, my experiences are, unfortunately, limited. I have found the form of broncho-pneumonia mentioned, and not the specific bronchial infective type seen in swine-plague, and which, considering the manner of infection, is of necessity by way of the intestinal canal, it is very doubtful if the bronchial infective variety ever occurs in connection with this germ, but I have seen another form of broncho-pneumonia in this disease due to an entirely adventitious bacillus capable of developing in the catarrhalic infected bronchioles, but which had no more connection with the disease in question than if the animal had been afflicted with verminous bronchitis, and exposed to dust of some kind in which such a germ was suspended. The occurrences are more common in the septicemic diseases of our domestic animals than any observers of this country or Europe seem to be aware of. In sections of such lungs, sometimes one variety and sometimes several varieties of adventitious germs may be found mixed up with the genuine organism, which latter may have had nothing whatever to do with the lesions in question. Ultra-bacteriological investigators go on a wild-goose chase after such adventitious germs, while men of pathological common sense cast away such chaff and pay their attention to the disease as it is, valuing complications as they should be appreciated. In this disease, as in swine-plague, we may frequently find an entire absence of the consolidation in the lungs, if I can judge by the very meager notes sent me on the lesions observed, but still more by a correct appreciation of the natural pathological results in such a disease. In the same way with the spleen : sometimes it is swollen, often excessively, at others not, but never-failing lesions will be those of a hematogenic character varying from capillary to coarse vascular engorgements and hemorrhages of various dimensions. The diffuse capillary engorgement of the kidneys, which may be considered as pathognomonic of the southern cattle-plague never occurs in this disease, and hence may eventually prove of essential differentio-diagnostic value, should it be finally shown that the germ of the corn-fodder also invades grasses, and causes disease in grazing animals in the Summer months. We shall allude to this question again later on. Acute lesions that will never be missed, aside from the disturbances of the circulation, are those of the dense parenchymatous glandular organs which are essentially specific to the extra-organismal septicemia, and vary in degree from clouded swelling to the most extreme grade of fatty degeneration, as has been shown in the very brief necroscopical notes.

To sum up, then, the corn-fodder disease is an *acute extra-organismal septicemia, due to a microörganism belonging to the class of ovoid, belted germs, to which variety of diseases also belong the swine-plague, southern cattle-plague, wild seuche, hen cholera, and yellow fever in man, but in no case are these micro-etiological organisms one and the same, but each is a specific entity capable only of causing its specific disease under natural conditions, in those animals specifically predisposed to its action from some utterly unknown but equally specific physiological idiosyncrasy peculiar to its species.*

### SYMPTOMATOLOGY OF THE CORN-STALK DISEASE.

In endeavoring to portray the symptoms of this disease, we come face to face with a question of exceeding difficulty, because of their very close resemblance to at least two other diseases which occur in cattle in our Western States, and even anthrax itself offers extra vital phenomena which very often more or less closely resemble those presented by this disease, especially in its most acute forms. This fact has led many veterinarians into most serious errors in diagnosis.

In the first place, as to duration, like the swine-plague, this disease may be fatal in twenty-four hours, or it may extend to eight or ten days before such a result occurs. It is not a universally fatal disease, and it is highly probable also of a non-recurrent type.

In the first place, we have to do with an acute blood-poison disease, which, like all such diseases, is accompanied by a more or less excessive exacerbation of the bodily temperature varying, so far as known, from 39° C. to 42° C., that is, from 102.2° to 107.6° F. Such an excessive rise in the temperature must necessarily be followed by equally severe changes in the parenchymatous organs of the body and consequent disturbances of the circulation, which frequently leads to excessive circulation, changes in the lungs, often followed by pneumonia and most insufficient oxidation of the blood; hence, under such circumstances, one would naturally expect a much accelerated and often very weak pulse and increased respiration, which, when consolidation of the lungs is present, is also very labored. (For a fuller account of these disturbances see my report on swine-plague.)

These disturbances of the circulation frequently extend to the brain, where engorgement and cerebral pressure occurs, which, in some animals, takes on the form of "craziness," as the owners call it. The animals then bellow fearfully and chase other animals, especially dogs, hogs, or fowls, but seldom human beings. This has led to the mistake of their being called "mad" at times, and to this disease being mixed up with anthrax, an entirely different one, by some veterinarians. Other animals stand by themselves, or are depressed and loth to move. Separation from their companions is one of the first indications of illness. As nearly as I can discover, they can all swallow and all drink. This is a very important point to be remembered. As in swine-plague, constipation is a very frequent occurrence, while laxity of the bowels also often occurs, and may be looked upon as a most favorable complication. Red urine does not occur. The visible mucosæ are injected, and often have a yellowish-red tinge. It will be remembered that my inoculated steer drank all the time, and even ate a little during its most ill days. That such sick animals should be disinclined to eat and often to drink, is no wonder, but if clear water is placed before them, no difficulty in swallowing will be discovered. Milch cows soon slacken in their yield of the lacteal fluid, frequently the secretion ceasing altogether for a time.

### DIAGNOSIS.

It has already been suggested that this disease cannot be mistaken for the southern cattle-plague. First, because of the season of the year in which the latter occurs, and the absence of Texas cattle. Second, the longer of approaching illness and the general average of its duration, as well as the non-existence of hematuria, red urine, in the corn disease, and the fact that the outbreak in the latter extends more slowly over a herd, and lasts longer under ordinary circumstances, or, in other words, the certainty of exposure to infection of the majority of the animals in the same herd at one and the same time is much greater in the southern cattle-plague than in the corn-fodder disease.

That it bears no relation to the wild seuche of Germany, is shown by the time in which it occurs, the locality and the circumstances upon which, or under which, it occurs, and the absence of enormous edema.

In fact, *when cattle, horses, or other herbivorous animals become*

*unaccountably ill, immediately after having been turned into a stripped corn-stalk field, and that illness is accompanied by the phenomena previously detailed, it may be taken for granted that it is the corn-fodder disease, and no other.*

PREVENTION OF THE DISEASE IN CORN AND OTHER FODDER.

Were we only in possession of that practical knowledge by which we could invariably tell when the growing corn in our fields was infected with this germ, so dangerous to our live-stock, in those the prevention would be the simple matter of avoiding such corn-fields as places for turning in stock for the past harvest gleaning. From conversation with several farmers, I am quite convinced that even now some of them have quite distinct ideas of the manner in which the corn itself is affected, and that we have only to obtain an exact description of this part of the story from the botanical side to be enabled to totally prevent this disease in our live-stock, so far as the gleaning of our corn-fields is concerned. But this leaves us to face several uncertainties which can only be settled by careful observation by farmers, and exact investigation by competent investigators.

FIRST.—Knowing that the corn fodder is diseased, the question is, Does the germ also penetrate the cob and growing kernel, and can they also cause the disease?

My own opinion would be to doubt it, had I not had very singular experience with a single cob of half-grown corn, and which at the time I did not have suspicion enough to investigate, and was also very busy in other directions and equally annoyed by the owner of a horse which ate some of this corn, so it was said, and it was assumed that the corn has been poisoned by a vicious neighbor. All that was brought me was a "nubbin" ear of half-ripe corn. This was fed to a rabbit, and the animal died three days afterwards. There was every indication of a blood-poison disease. Anthrax was looked for, but not present. The organs were taken by the owner to a chemist, but nothing found therein. Cultures were not made, and only a very casual examination of the blood for bacillus anthracis, as I was busy beyond endurance at the time, and have never had any assistant to whom I could turn over the many such cases which have come to my notice.

This case is simply quoted as a warning worthy of the attention of other investigators.

SECOND.—Are grasses also infected by this germ, and if so, which?

This last is a very important question, for if they cannot be, then such fields can be used for raising grasses. This can be best determined by actual experiment by intelligent farmers who should turn such fields into grasses, including patches of millet and clover, and then feed a few cattle with each kind without the admixture of any other herbaceous food.

It would be well if the experiment stations would make suitable arrangements in this direction with intelligent farmers, and bear the expense of using such infected corn-fields for experimentation in about the following manner:

First—It should be thoroughly cleansed of all refuse of the old corn-crop, but not exposed to the action of fire.

Second—A portion should be planted in corn, and if any stalks appear diseased during the growing period, they should be fed experimentally, and under every precaution against accident from other causes, to cattle and also rabbits. The latter might be tried first, as enough is now known of the germ of this disease to enable any competent person to recognize it correctly. When the corn is ripe, the ears should be gathered carefully, and all full ears separated from those incomplete in development. Feeding experiments should be made with both. After the crop of corn has been gathered, a few cattle should be turned into it; and in order to avoid any error, exact methods should be taken to see that they were sufficiently supplied with water and salt.

Naturally, each section of the field should be separated from the other, and if, as Prof. Burrill's letter suggests, such a field as one of "forty acres" can be pretty generally infected, such a one should be used for this kind of experimentation.

Third—Various grasses, including millet and clover, in fact all kinds used for feeding stock, should be planted in lots in such a field; one of each kind should be used for pasturage in the Summer for a few cattle, while the crop of another lot should be preserved for Winter use, and then fed to a certain number of cattle.

It is too much to ask any farmer to do all this at his own expense, but the interests at stake make it the imperative duty of the agricultural experiment stations to do it—this being one of the purposes for which they were created, though Nebraska is the single State that has done its duty in this regard. There are an abundance of public-spirited and intelligent farmers who will gladly support the work. In fact, Mr. McKelvie has written me that he intends to follow out my suggestions as to grasses and millet, with the field he spoke of in his very detailed letter stating his experiences with the disease.

Wherever such a disease has occurred, every stalk and leaf left on such a field should be destroyed by fire, and, until we know to the contrary, the field should be seeded down to hay. There is no question but what the infected remnants of the corn-fodder can, upon their decay, cause the further infection of the field by the germ thus becoming free and again developing in the soil.

### PREVENTION OF THE DISEASE IN LIVE-STOCK.

In discussing this question, I will limit myself to cattle, because they are the only species of our domestic animals in which the disease causes serious loss; in fact, the only one in which we know to absolute certainty that it occurs.

Some might think it strange if I did not say a word about *treatment*, while my scientific colleagues might think it equally strange should I do such a thing, for medicinal treatment in any curative sense is the height of absurdity in any disease of this class; yet a close study of the clinical symptoms of this peculiar disease, and some knowledge of its pathology, does show that the offering of purgative doses of a saline character, Glauber salts, to cattle, in the very earliest stages of the disease, as well as to every member of a herd in which some have become ill upon exposure to this disease in a stalk-field, is a matter of prophylactic importance. No harm can certainly be done by the thorough cleansing out of the intestinal tract in the animals still undiseased, and as for those diseased, especially, and perhaps only those in the early stages, such a method of treatment is most certainly indicated as a possible means of equalizing the disturbances in the vascular system, and, perhaps, the avoidance of cerebral and pulmonary complications, as well as a tendency to deplete the blood of some of its septic elements, and to check the supply by the removal of so many of the specific poisonous elements as must necessarily occur in such a cleansing of the digestive tract.

Such treatment must, however, take place within quarantined limits, only so that all the manure and litter soiled thereby can be destroyed by fire when the outbreak is over. It must be borne in mind that if the manure and litter from a cattle-yard, where animals have had this disease, is taken out and strewed over a field and then plowed in, and that field is planted to corn, that such corn is very liable to become invaded by this germ and can thus be the cause of more losses in cattle if turned into such a "stalk-field" the ensuing Fall or Winter.

On the eruption of this disease in a herd of cattle which have been used to do the lazy man's gleaning in a stalk-field, the first step to be taken is the peremptory withdrawal of the herd from such a field and such fodder. The next thing to do is either to number, brand or adopt some other means by which a record can be kept of each animal in the lot, and then take the temperature of each one night and morning. All with a temperature of over 100° F. must be looked upon as suspicious, and those in which it exceeds 102° F. as diseased. Those in which it does not exceed or rise over 100° F. need not cause any worry. There is no need of separating the sick from the well, as the disease is not contagious. As mentioned previously, a saline purgative is indicated for all the animals. Those that die should be cremated, and with them a lot of the litter in the yard. If possible to avoid it, the regular cattle-yard should never be used for such cattle after any of them have become ill. Again, I repeat, in no case should a particle of the manure or refuse from a place where such cattle have been confined, ever be used for fertilizing purposes.

*Burn it up, as well as the animals which die.*

PATHO-BIOLOGICAL LABORATORY, LINCOLN, Neb., June 1, 1889.

